# Antimicrobial Properties of Selected Copper Alloys on *Staphylococcus aureus* and *Escherichia coli* in Different Simulations of Environmental Conditions: With vs. without Organic Contamination

**DOI:** 10.3390/ijerph14070813

**Published:** 2017-07-20

**Authors:** Anna Różańska, Agnieszka Chmielarczyk, Dorota Romaniszyn, Agnieszka Sroka-Oleksiak, Małgorzata Bulanda, Monika Walkowicz, Piotr Osuch, Tadeusz Knych

**Affiliations:** 1 Faculty of Medicine, Chair of Microbiology, Jagiellonian University Medical College, Czysta str. 18, 31-121 Kraków, Poland; agnieszka.chmielarczyk@uj.edu.pl (A.C.); d.romaniszyn@uj.edu.pl (D.R.); a.sroka88@gmail.com (A.S.-O.); mbulanda@post.pl (M.B.); 2 Faculty of Non-Ferrous Metals, Department of Metal Working and Physical Metallurgy of Non-Ferrous Metals, AGH University of Science and Technology, Al. A. Mickiewicza 30, 30-059 Krakow, Poland; mwa@agh.edu.pl (M.W.); piosuch@agh.edu.pl (P.O.); tknych@agh.edu.pl (T.K.)

**Keywords:** antimicrobial copper, hospital environment, *Staphylococcus aureus*, *Escherichia coli*, environmental disinfection, patient safety

## Abstract

*Background:* Hospital equipment made from copper alloys can play an important role in complementing traditional methods of disinfection. *Aims of the study:* The aim of this study was to assess the dynamics of the antimicrobial properties of selected copper alloys in different simulations of environmental conditions (with organic contamination vs. without organic contamination), and to test alternatives to the currently used testing methods. *Materials and Methods:* A modification of Japanese standard JIS Z 2801 as well as *Staphylococcus aureus* (SA) and *Escherichia coli* (EC) suspended in NaCl vs. tryptic soy broth (TSB) were used in tests performed on seven commonly used copper alloys, copper, and stainless steel. *Results:* A much faster reduction of the bacterial suspension was observed for the inoculum prepared in NaCl than in TSB. A faster reduction for EC than for SA was observed in the inoculum prepared in NaCl. The opposite results were found for the inoculum based on TSB. A significant correlation between the copper concentration in the copper alloys and the time and degree of bacterial suspension reduction was only observed in the case of EC. *Conclusions:* This study confirmed the antimicrobial properties of copper alloys, and additionally showed that *Staphylococcus aureus* was more resistant than *Escherichia coli* in the variant of the experiment without organic contamination. However, even for SA, a total reduction of the bacterial inoculum’s density took no longer than 2 h. Under conditions simulating organic contamination, all of the tested alloys were shown to have bactericidal or bacteriostatic properties, which was contrary to the results from stainless steel.

## 1. Introduction

There are different reservoirs of micro-organisms in a hospital’s inanimate environment [1,2,3]. The proper use of hand hygiene by health care workers is a proven method of patient protection from pathogenic micro-organisms that are commonly present in the hospital environment [4]. However, despite the intensification in recent years of educational campaigns devoted to hand hygiene, it is still commonly skipped or incorrectly performed, especially in countries with a limited timeframe for infection control in modern practice [5,6,7]. Reports indicating a reduced susceptibility to disinfectants and antiseptics in healthcare settings can also be found [8]. In this situation, placing equipment/touch surfaces made of materials with antimicrobial properties may be a way of complementing the traditional methods of disinfection. Copper alloys are one of the materials with antimicrobial properties. Although the antimicrobial properties of copper and its effectiveness in hospital room equipment have been confirmed in some clinical studies [9,10], there are still few hospitals or healthcare centres that utilize it. Gorman and Humphreys analysed the advancement and degree of employing copper as a material with antimicrobial properties in preventing and controlling infection. They concluded that it is a field that still requires further research in a number of areas before the widespread implementation of copper contact surfaces can be recommended [11]. Gouldi et al. demonstrated that antibacterial activity (especially the time of total bacterial reduction) differs not only between bacterial species but also within specific species [12]. The rate and extent of the elimination of micro-organisms exposed to copper alloys is highly dependent on the parameters of the experiment [13]. A certain disadvantage in contact surfaces composed of materials based on copper is their susceptibility to corrosion from the sweat of human hands, which, together with continuous use, significantly decreases the products’ aesthetic value. Additionally, many people consider the silver sheen typical of stainless steel products to be associated with sterility and hence microbiological safety. Airey and Verran draw attention to problems associated with cumulative soiling and cleaning, consisting of the fact that organic soiling has a better affinity for products containing copper than for stainless steel (SS) [14].

As a result, work on producing copper alloys with optimal antimicrobial and performance properties is being conducted, and an indispensable stage in this type of research is to determine the antimicrobial properties of the individual materials.

Studies on the antimicrobial properties of copper and its alloys have been conducted using various methods [15]. The number of parameters influencing the results obtained has an almost infinite set of combinations of microbial suspension densities, exposure times, and additives to the suspension, with the aim of simulating different types of exposure (e.g., organic contamination) on microbes in the environment. In the experiments performed to date, two groups of methods were used: dry and wet exposure; numerous variations of these methods have been employed by researchers. For example, formal recommendations are also based on different approaches: the Environmental Protection Agency (EPA)’s recommendations are based on the dry method of exposure, whereas the Japanese recommendations are based on the wet method [16,17]. Both have advantages as well as disadvantages. In both protocols, *Staphylococcus aureus* and *Escherichia coli* (different strains) are used to test antimicrobial properties, and the EPA’s recommendations also include *Pseudomonas aeruginosa* and *Enterobacter aerogenes*. From a broad epidemiological approach, *Staphylococcus aureus* (SA) and *Escherichia coli* (EC) are the most important. Methicillin resistant SA is a major cause of healthcare-associated infections in Europe [18], and is a common cause of out-patient infections of different types [19]. EC constitutes not only an important cause of healthcare-associated infections, but is also a leading cause of diarrhoea in humans and has an important impact on public health [20,21], which leads researchers to undertake studies of different ways of preventing infections of that aetiology [22].

The aim of this study was to assess the dynamics of the antimicrobial properties of selected copper alloys on *Staphylococcus aureus* (ATCC 12493) and *Escherichia coli* (ATCC 25922), as representatives of important infection control and public health Gram-positive and Gram-negative bacteria, in different simulations of environmental conditions: with organic contamination vs. without organic contamination (bacteria suspended in NaCl vs. tryptic soy broth TSB). Additionally, the aim of this study was to test alternatives to the currently used methods of testing the antimicrobial properties of non-porous material for specific application in healthcare settings.

## 2. Materials and Methods

### 2.1. Chosen Copper Alloys and Their Preparation

Metal samples measuring 2.5 cm × 2.5 cm with a thickness of 1–2.5 mm were provided by the Faculty of Non-ferrous Metals, AGH University of Science and Technology, Kraków. Before their delivery for microbiological testing, the samples underwent mechanical polishing, cleaning, and degreasing by immersion in acetone. Prior to use for microbiological testing, the samples were sterilized by wiping with 96% alcohol. Studies were conducted on the following copper alloys: brass (CuZn37, CuZn15), tin bronze (CuSn6), aluminium bronze (CuAl10Ni5Fe4), copper-nickel alloys (CuNi10Fe1Mn), nickel silver (CuNi12Zn20, CuNi18Zn20), and for ETP copper (99.9% Cu) as a positive control (presumed highest antimicrobial efficacy) and stainless steel as a negative control (assumed lack of antimicrobial properties). The copper alloys selected for this study are the most well-known and most frequently used in various industries. The alloys used in the study, with data on the concentration percentage of copper, are listed in Table 1.

### 2.2. Quantitative Culture Method to Determine the Antimicrobial Effectiveness of Copper and Its Alloys

In this study, wet exposure was used (modified methodology of the Japanese Standard [17]), and is described in detail below, to assess the antimicrobial properties of the selected copper alloys.

Antimicrobial efficacy testing was conducted using a bacterial suspension of *S. aureus* (SA) and *E. coli* (EC) prepared in saline (NaCl) and tryptic soy broth (TSB, BIOCORP, Warsaw, Poland) according to the formula described below. Bacterial suspension in NaCl was chosen for simulating environmental conditions without organic contamination and TSB as a medium for bacterial suspension was the option for simulating an environment contaminated with organic compounds (TSB includes caseine and soy peptone and glucose in proportions optimal for bacterial growth).

The bacterial strains to be tested were stored in glycerol at −70 °C. One day before antimicrobial efficacy testing, a small amount of the suspension was taken from a frozen sample, inoculated onto solid Muller–Hinton agar (MHA, BIOCORP, Warsaw, Poland) (clean culture) and then incubated for 24 h at 37 °C. From the culture obtained, a suspension was prepared in saline at a density of 0.5 McFarland standard (controlled using a densitometer bioSan, Riga, Latvia). Subsequently, 100 µL of the suspension with a density of 0.5 McFarland standard was transferred to 900 µL of saline; the test inoculum version was a clean bacterial inoculum with no additives or 900 µL of TSB; the test inoculum version was based on a suspension simulating organic soil that is favourable for the multiplication of bacteria. Each time, a check was performed on the viability of the bacteria obtained in the culture on solid medium and the control of the precise initial concentration (its density is expressed in CFU/mL).

Samples of the metals (coupons) tested were placed in a sterile container made of PVC with a capacity of 100 mL that was 6 cm in diameter, and then 100 µL of the test suspension was applied (the composition depended on the variant of the experiment). Next, the coupon was covered with sterile polypropylene foil measuring 2 cm × 2 cm to provide close contact between the bacterial suspension and the metal surface. The container was covered to prevent contamination of the sample with microbes from the air, but it remained loose enough that aerobic conditions were maintained throughout the course of exposure and when left for a specified period of time (0, 15, 30, 45, 60, 90, 120, 180, 240, and 300 min) at approximately 22 °C (room temperature).

After a certain period of time, 5 mL of the TSB (tryptic soy broth, BIOCORP, Warsaw, Poland) solution and approximately 30 sterile glass beads that were 2 mm in diameter were placed into the container and shaken for 2 min in a shaker (shaker-incubator ES-20/60, Riga, Latvia). Then, 100 µL of the wash was collected, and four serial decimal dilutions were prepared, of which 100 µL was inoculated onto solid MHA for each time-point. After a 24 h incubation, individual colonies were counted on the plates when the resulting number was countable.

For each metallic material, each exposure time for both microbes was repeated three times. To count the amount of CFU/mL after the exposure of the bacterial suspension to the studied materials, the average of the triplicates was used. The formula for the calculation was CFU/mL = (n × f × v_1_)/(v_2_ × v_3_), where: n is the average number of colonies/plate in dilution, f is the dilution factor, v_1_ is the volume of TSB used for washing the bacteria that survived after exposure, v_2_ is the volume used and applied on metallic coupons, and v_3_ is the volume of the plated material (v_1–3_ in mL).

To evaluate the effectiveness of the antimicrobial activity, the criteria used by Souli et al. [23] were adopted, according to which a suspension density reduction occurred, ranging from ≤2 to <3 log mean bacteriostatic properties, as well as a reduction of over 3 log bactericidal properties. Ole et al. estimated that the microbiological load of touch surfaces, such as door handles in hospital wards, was 1–6 × 10^3^ CFU [24], and therefore the criteria proposed by Souli et al. can be deemed to be proper for testing the antimicrobial efficacy of products made of copper and its alloys. According to the EPA protocol, when there is an observed reduction of more than 99.9% of pathogen cells on a specific product, it can be considered to be a sanitizer [16]. For a starting bacterial density of approximately 1 × 10^7^ CFU/mL, this would indicate a 3 log final reduction (to 1 × 10^4^ CFU/mL).

### 2.3. Microscopic Assessment of the Degree of Reduction in Live Bacteria on Metallic Materials

For the selected materials, the copper alloys (Cu-ETP, CuZn37, and CuSn6) with the highest antimicrobial activity and/or those that are commonly applied in practice, and for stainless steel, an additional test was performed using a microscopic technique.

The following strains were used in this study: *Escherichia coli* ATCC 25922 and *Staphylococcus aureus* ATCC 12493. Precultures were prepared for the experiment from the same stock culture by streaking the stock solution onto Columbia agar plates. After 18 h of incubation at 37 °C, several colonies were transferred into 10 mL of NaCl. The optical density was measured using a densitometer (bioSan, Riga, Latvia). Each time, a bacterial suspension was used for the experiments with a 0.5 MF optical density. Then, 100 µL of the bacterial suspension was transferred onto the tested material with dimensions of 2.5 cm × 2.5 cm and covered with sterile foil. Then, the prepared samples were incubated at room temperature for the following time periods: 15 min, 30 min, 60 min, and 180 min. After a predetermined time, the bacterial suspension was collected from the materials using disposable pipettes and a CytoOne cell scraper (Starlab) and transferred to Eppendorf Tubes. The staining procedure was conducted using a LIVE/DEAD BacLight Bacterial Viability kit L13152 (Molecular Probes, Thermo Fisher Scientific, Eugene, OR, USA).

The SYTO 9 stain generally labels all of the bacteria in a population (both those with intact membranes and those with damaged membranes). By contrast, propidium iodide only penetrates bacteria with damaged membranes, which causes a reduction in the SYTO 9 stain fluorescence when both dyes are present. Thus, bacteria with intact cell membranes stain fluorescent green, whereas bacteria with damaged membranes stain fluorescent red. Stock solutions of each dye were prepared individually and according to the manufacturer’s instructions [19]. After staining, the preparations were assessed by fluorescence microscopy (Olympus) with two filters, FITIC and TRITIC, at 100× magnification with the use of immersion.

## 3. Statistical Analysis

The means of three repetitions for each material, exposure time, and bacterial species were used in the statistical analyses.

The antimicrobial activity over time is presented on scatter plots with a log scale. For better visualization, values of less than one were presented as having a value of one. The best curve describing the antimicrobial activity shape of the studied bacteria’s substrate over time and on different materials was fitted using a command curvefit from STATA IC 13 for Windows, which estimates the fitting parameters among a wide variety of functions and is based on the highest R2. Finally, the geometric (Y = b0X b1X), exponential (Y = b0exp(b1 X)), and rational (Y = (b0 + b1X)/(1 + b2X + b3X2)) functions’ families were chosen and presented. Additionally, the associations between the amount of Cu in a particular material, the time of reduction to the level of “0”, and the percentage decrease of the bacterial amount over time (to the beginning value) were estimated using Spearman rank correlation. The statistical significance was established at *p* < 0.05.

The datasets used and/or analysed during the current study are available from the corresponding author upon reasonable request.

## 4. Results

The results are presented in the figures. In all of the figures, points represent real values of the calculated CFU/mL. The solid lines show the fitting mathematical functions for the real values. The formulas of the fitted functions for all tested metallic materials and both bacteria species are given in Table 2.

**Figure 1:**
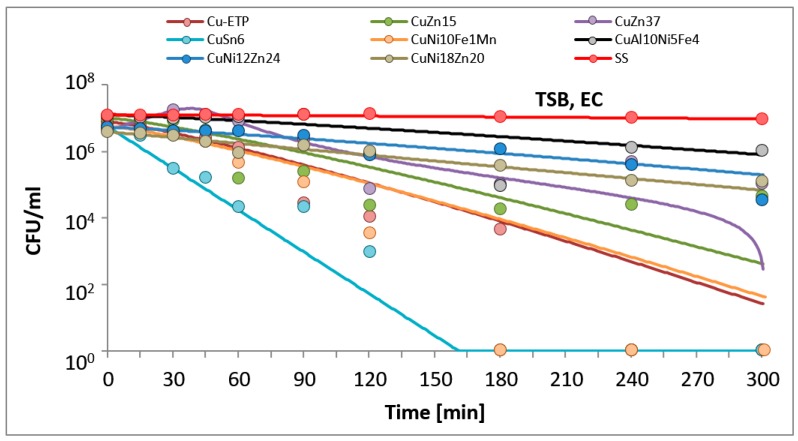
*Escherichia coli* (EC) suspension density (CFU/mL) reduction on tested metallic materials, in the variant of the experiment simulating organic contamination. TSB, tryptic soy broth.

**Figure 2:**
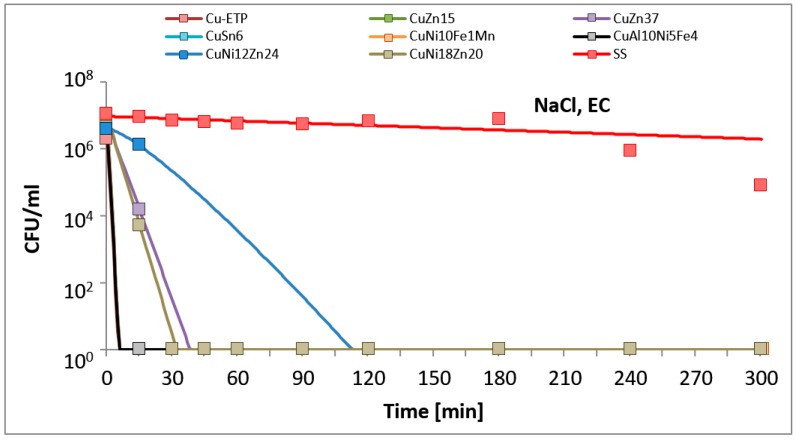
*Escherichia coli* (EC) suspension density (CFU/mL) reduction on tested metallic materials, in the variant of the experiment simulating lack of organic contamination.

**Figure 3:**
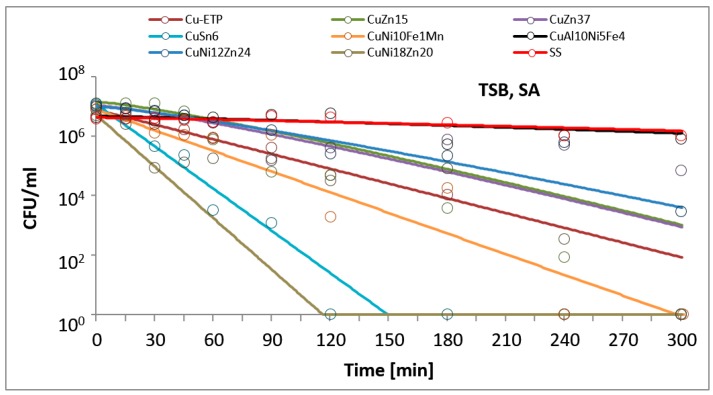
*Staphylococcus aureus* (SA) suspension density (CFU/mL) reduction on tested metallic materials, in the variant of the experiment simulating organic contamination. TSB, tryptic soy broth.

**Figure 4:**
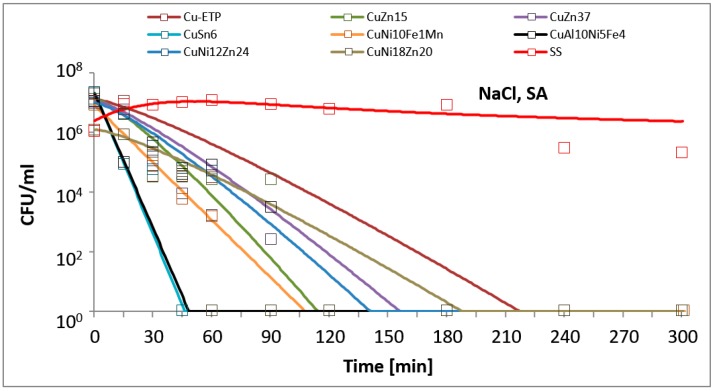
*Staphylococcus aureus* (SA) suspension density (CFU/mL) reduction on tested metallic materials, in the variant of the experiment simulating lack of organic contamination.

The time and degree of bacterial suspension density reduction depended on the type of inoculucum (in NaCl vs. TSB) and on the bacterial strain. However, the best antimicrobial properties against the studied EC and SA reference strains were found for Electrolytic Tough Pitch (ETP) copper and for tin bronze CuSn6, regardless of the composition of the suspension used for testing. Significant differences were observed in regard to the suspension’s composition. All of the tested alloys demonstrated increased antimicrobial activity against both EC and SA suspended in NaCl compared to the suspensions in TSB. A reverse regularity was observed for the bacterial inoculum in TSB: for all of the copper alloys tested, the EC strain tested was less sensitive than SA. Detailed test results are presented in Figure 1, Figure 2, Figure 3 and Figure 4. The time needed for a complete density reduction in the EC bacterial suspension in NaCl was significantly shorter (15 min) for alloys that had a copper concentration of more than 64% in comparison with alloys with copper concentration from 62 to 64% (30 min); *p* = 0.008. A complete reduction after 15 min was observed for Cu-ETP, CuZn15, CuSn6, and CuNi10Fe1Mn as well as after 30 min for CuZn37, CuNi12Zn21, and CuNi18Zn20. A significant correlation between the concentration of copper in the alloy and the time needed to obtain a complete reduction in the suspension density in NaCl was not shown for SA (*p* = 0.688). The time needed for a complete reduction in the SA suspension in NaCl ranged from 45 min for CuSn6 to 120 min for CuZn37 and CuZn15 (Figure 4). Additionally, stainless steel demonstrated a slight reduction in the density of the initial EC and SA suspensions in NaCl, but lower than two logarithms. However, this degree of reduction does not allow the bacteriostatic properties to be confirmed from the criteria assumed.

In the experiments simulating environmental conditions contaminated with organic compounds (suspension in TSB), significant differences were also observed in regard to EC, for which a complete reduction in a time between 180 and 240 min was obtained for alloys that had a copper concentration of over 85% (*p* = 0.021), and no significant correlation was found for SA (*p* = 0.088). Better antimicrobial properties in this case were observed against SA than EC for all copper alloys, contrary to the stainless steel, which did not show a decline in suspension density in this case (Figure 1 and Figure 3). The greatest antimicrobial activity in the TSB suspension was found for Cu-ETP, CuSn6, and CuNi10Fe1Mn, in which SA and EC both showed a complete reduction in the density of the initial suspension after, at most, 240 min (Figure 1 and Figure 3). CuZn37 demonstrated the lowest antimicrobial activity, because after 300 min of exposure, both SA and EC showed bacteriostatic effects. In the cases of CuNi18Zn20 and CuZn15, a complete reduction after 300 min was only found for SA; the CuNi18Zn20 material did not demonstrate bacteriostatic properties against the EC strain after this time, whereas CuZn15 did show a reduction of approximately 2.5 log after 300 min, and therefore bacteriostatic properties.

**Figure 5:**
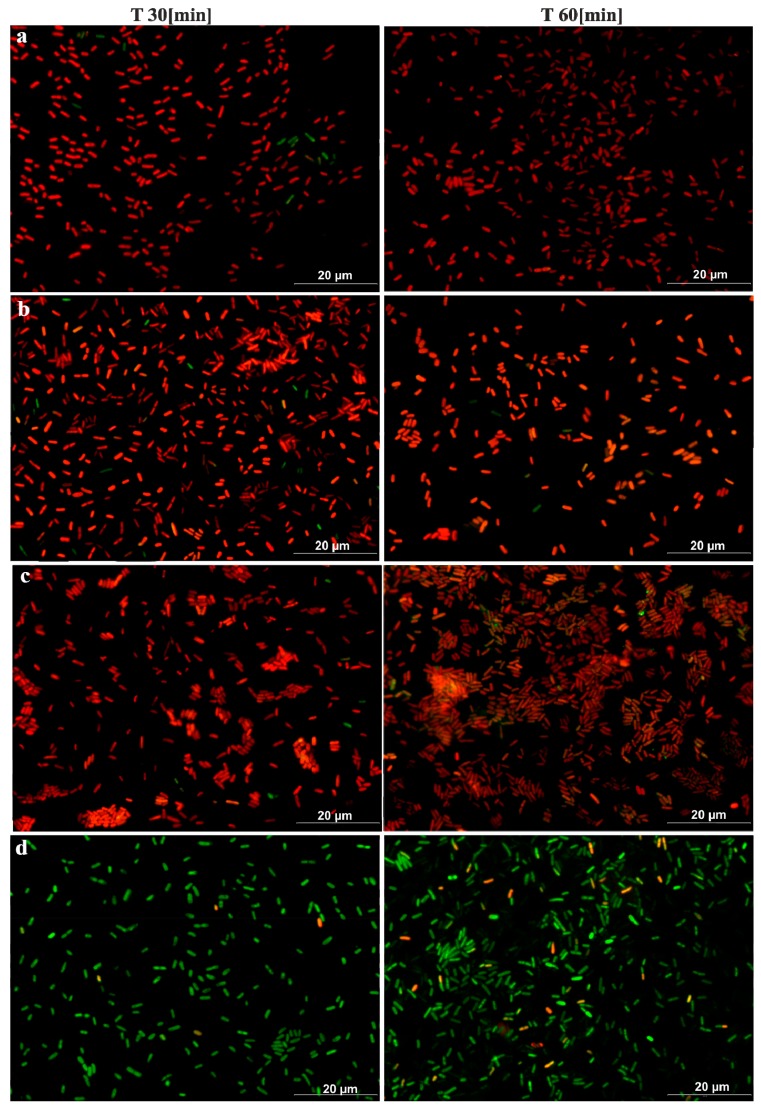
Photographs showing the survival rate of *E. coli* in selected time periods on: Cu-ETP (**a**), CuZn37 (**b**), CuZn6 (**c**), and stainless steel (**d**).

**Figure 6:**
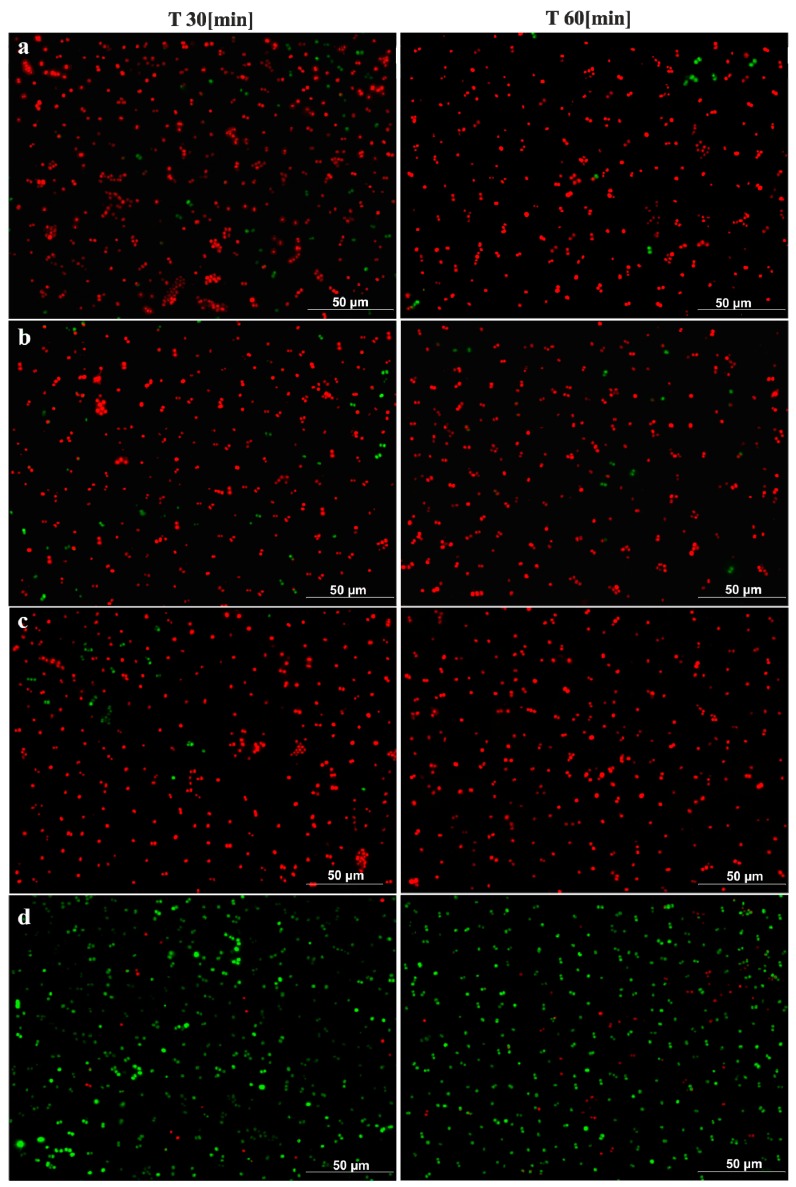
Photographs showing the survival rate of *S. aureus* in selected time periods on: Cu-ETP (**a**), CuZn37 (**b**), CuZn6 (**c**), and stainless steel (**d**).

The antimicrobial activities of Cu-ETP, CuZn37, and CuSn6 was confirmed with microscopic tests. After 15 min, a considerable reduction of EC and a partial reduction of SA were observed on the surface of the aforementioned copper alloys, whereas after 30 and 60 min, over 90% and 95% of the bacteria, respectively (both *E. coli* and *S. aureus*), were killed (or had incompetent membranes). However, no reduction was observed regarding the EC and SA viability on the stainless steel surface, even after 180 min. The results of the fluorescence analysis (Figure 5 and Figure 6) were similar to the survival curves (Figure 2 and Figure 4).

## 5. Discussion

This study was conducted as part of a project devoted to assessing the antimicrobial properties of copper and its alloys for application on touch surfaces in healthcare centres. One of the key aims of the study was to evaluate the properties of copper alloys subjected to various types of chemical, physical, and mechanical modifications. To assess the antimicrobial properties of hard, non-porous materials, the guidelines of the Japanese Standard are available [17], and a range of copper alloys that are approved for use have been evaluated using the EPA’s methodology [16]. Both of these documents adopt strictly fixed points in time after which antimicrobial efficacy is examined: after 24 h in the case of the Japanese Standard, and after 2 h in the case of the EPA’s recommendations. However, in healthcare settings, most touch surfaces undergo cleaning or disinfection at least once per day. Subsequently, over the course of 24 h, there are many occasions to transfer the microbes that inhabit such surfaces on the staffs’ hands to patients susceptible to infection. As a result, recommendations for the antimicrobial testing of products designed for touch surfaces in healthcare should take into account the shorter time period. Similar theses were posed by Gorman and Humphreys [11]. Therefore, the assumption adopted in this study was to determine the shortest possible time required for the anti-bacterial activity and conditions to be increased/decreased. We used a modification of the Japanese Standard, because the ISO 22196:2011 standard for antimicrobial testing based on it is recommended and practically applied in Europe. The other reasons for using this standard were the possibility of comparing our results with the results of other researchers published in the literature, and because wet exposure represents an environment that is more conducive to bacteria.

The results obtained in this study are consistent with the results obtained by other authors in terms of confirming the stronger antimicrobial activity of copper in comparison with its alloys [25,26,27,28,29,30]. However, in this study, differences were observed regarding the rate of reduction in the initial EC and SA bacterial suspensions in comparison with studies by other authors. Gouldi et al. tested the antimicrobial properties of copper against different strains of SA, methicillin-resistant *Staphylococcus aureus* (MRSA), and EC as well as other species [12]. The authors found a complete reduction in the initial suspension density of the tested EC and MRSA strains from 40 to 80 min, depending on the strain. A complete reduction in the initial density of the EC suspension on TSB (from the level of approx. 10^7^ CFU/mL) in the present study was observed for only three alloys, and for the other four alloys, a partial reduction was demonstrated (bacteriostatic or bactericidal properties). However, in the case of the SA suspension, a complete reduction was observed for five out of seven of the alloys examined. In a study by Gouldi et al., 20 µL of the suspension was placed on metal samples without any cover [12]. In our study, five times this volume was used, but the application of foil on a drop of the applied suspension also ensured that there was a proportionally larger metal–bacterial suspension coverage area. However, there were also some differences regarding the preparation of the metallic coupon. In the study by Gouldi et al., the metallic coupons were cleaned and degreased very thoroughly (including vortexing in 10 mL of acetone containing 30 glass beads; in the protocol of the EPA standard, the metallic coupons are additionally physically soaked for 2–4 h in a detergent) [12,16]. In our experiment, after polishing, the coupons were simply immersed in acetone for one minute. The way of the coupons’ preparation implies the degree of wettability, which translates into the adhesion of bacterial inoculum (bacterial cells): the more wettable the surface is, the more close contact there is and the faster damage occurs to the bacterial cells.

However, a much faster and complete reduction in this study was observed for all of the tested materials made of copper alloys for EC and SA in the case of a suspension prepared with NaCl. Souli et al. [23], who investigated the bactericidal efficacy of copper and brass (CuZn37) in comparison with stainless steel and PVC on Gram-negative rod-shaped bacteria that produce beta-lactamase, obtained partially similar results for EC to the results presented in this study for the variant of the experiment using a TSB suspension. For CuZn37, after 300 min, they found a reduction in the initial suspension of 2 log, and for Cu, they found a reduction of slightly more than 2 log [23]. In this study, in regard to the exposure of the EC suspension on copper, a complete reduction in the suspension density was demonstrated after 240 min. An investigation by Waever et al. showed a complete reduction of the SA suspension density for pure copper at 1 h after inoculation; however, the examination was performed using the “dry” method [28]. Contrasting findings (a faster total bactericidal effect) were also obtained by Noyce et al., who examined the behaviour of three different methicillin-resistant *Staphylococcus aureus* strains [26]. The authors found a complete reduction in the initial suspension density from a level of approximately 1 × 10^7^ to zero in a time no longer than 90 min for copper (C19700), and for two MRSA strains on bronze (C24000), they observed a complete reduction after approximately 4.5 h. For one of the strains tested, the degree of reduction after 360 min did not meet the qualifying criterion for bacteriostatic properties [26].

Steindl et al. also showed a different dynamic of reduction in a bacterial suspension of Gram-negative rod-shaped bacteria and *Staphylococcus aureus* on copper than that observed in our experiment, although they prepared their bacterial suspension on TSB and similarly transferred 100 µL of the investigated suspension [31]. For New Delhi metallo-beta-lactamase-1 (NDM-1) producing *K. pneumoniae* and extended spectrum β-lactamase (ESBL)-type cefotaxime-resistant-Munich (CTX-M) producing *E. coli*, the authors found a complete reduction in the suspension density from approximately 1.5 × 10^8^ to zero as well as for MRSA after two hours (an opposite result to that observed in our study).

To date, the results of the published microscopic examinations analysing the survival of bacteria on copper surfaces have mainly been concerned with dry exposure, and are characterised by large differences regarding the time after which there was contact killing. In the study by Santo et al., a 90% or complete reduction in EC vitality was observed after only 1 min of exposure on copper surfaces [32]. Other authors observed such effects after only 45 min [33]. The latter results, despite dry exposure, are the closest to the results obtained by our study. Some authors claim that wet exposure may lead to longer survival of bacteria on copper [32]. However, in the study by Bleichert et al., and in the case of wet exposure, the time needed to kill various bacterial species ranged from 30 s for *Burkholderia mallei* and *Burkholderia pseudomallei*, to 1 min for *Y. mallei* and *B. pseudomallei*, and 3 min and 5 min for *Brucella melitensis* and *Francisella tularensis*, respectively [34]. The reason for the short survival, regardless of the species of bacteria, may be fixation with formaldehyde immediately before staining. However, the authors performed a control experiment using *E. coli* that did not demonstrate a negative impact of formaldehyde on staining, and the procedure of fixing itself, which requires additional centrifugation and washing steps, shifted the ratio of bacteria towards dead bacteria [34]. Therefore, the preparation of the material for staining and microscopic observation should be conducted with no additional activities or compounds that could distort the subsequent interpretation of the results. A partial limitation of staining is also the necessity of applying a neutral medium. Data from the literature emphasise that it is likely that media that are rich in growth factors (such as the TSB broth) can react with dyes, which leads to under- or over-estimations of the number of living cells, which in turn results in misguided conclusions [35,36]. For this reason, the tests for the microscopy part were conducted only with bacterial suspension in NaCl.

Based on the research presented in our manuscript, we are not able to clearly state or exclude the influence of the antibacterial copper mechanism on the integrity of the bacterial cell membrane. It is possible that copper can “kill the cell” by breaking the cell membrane, but is possible also another mechanism—by weakening the cells, and the use of dyes—can “help” break the membrane. In the available literature, there is insufficient information on this point. Confirmation or refutation of the above speculations may be possible using an electron microscope.

The conclusions resulting from this experiment may have practical applications concerning standards for testing antimicrobial non-porous materials, especially regarding various types of metals. Currently, both the Japanese Standard and the EPA’s recommendations [16,17] consider specific (disparate in both documents) patterns of conduct. However, standards regulating the testing of the antimicrobial properties of chemical disinfectants anticipate different variants of examination for specific applications (medicine, veterinary medicine, food industry), simulating various conditions (with and without organic contamination).

Bacterial contamination of touch surfaces in healthcare facilities can vary significantly between countries, hospitals, or even wards [3,37]. In a recent Polish study on the contamination of touch surfaces in different hospital wards, the growth of isolated bacteria was not abundant and *Staphylococcus* was the dominant genera in most samples [37]. Different results were obtained by Garcia-Cruz CP et al. in Mexico, who reported that Gram-negative rods were commonly isolated from the indoor hospital environment [3]. Oie et al. estimated the microbiological load of touch surfaces such as door handles in hospital wards to be 1–6 × 10^3^ CFU/mL, whereas Souli et al. suggested a criterion for reducing the bacterial suspension density by at least 3 log_10_ for bactericidal activity [23,24]. Such a result was demonstrated in the case of contamination without organic contamination for all of the copper alloys tested (no longer than 30 min for EC and 120 min for SA), which may find applications not only in the healthcare sector but also in other public utility units (such as public transport, schools, and kindergartens). The results of our experiments with bacteria suspended in TSB were weaker, but still, most of the tested alloys proved to have satisfactory antimicrobial properties. However, it is also worth mentioning that the conditions of our experiments were more severe than those applied in the American (in the American protocol: dry exposure with a five times smaller inoculum volume and a different way of preparing the metallic carriers) and Japanese standards (exposure time in the Japanese standard was less appropriate for simulation of the real world). In our experiment, under simulated conditions with organic contamination, we confirmed the bactericidal or bacteriostatic properties for the copper alloys tested, contrary to stainless steel, for which no reduction in the bacterial density was observed.

Additionally, we hypothesised that there is a pattern of microbial reduction rate which could be described by some family of mathematical functions. Whereas microbial reduction in time in the case of suspension in NaCl estimated by exponential or geometric function families presented fairly good fitting, the case of TSB had worse fitting parameters as well as different patterns of activity over time. To conclude, it was not possible to indicate a satisfactory approximation of the microbial reduction function in the case of some materials, which may indicate that it may not be easily predictable.

Despite this, our results confirm the bactericidal or bacteriostatic properties of the copper alloys tested.

## 6. Conclusions

1. The study confirmed the bactericidal or bacteriostatic properties of the copper alloys tested, in both variants of the experiment: with vs. without organic contamination.
2. A statistically significant correlation between the copper concentration in the alloy and the level and time of bacterial density was observed only for the *Escherichia coli* tested strain, and not for *Staphylococcus aureus.*
3. In the case of *Staphylococcal aureus*, the copper alloys tested showed better antimicrobial properties in the environment simulating organic contamination, but the survival time for this tested strain was longer in the conditions simulating no organic contamination compared to *Escherichia coli*.
4. The use in hospitals of equipment made of copper alloys should help to prevent the spread of pathogenic micro-organisms.

## Figures and Tables

**Table 1:** Compositions (%) of the tested commercial copper alloys.

Common Name	UNS * Code	Cu	As	Bi	Cd	Fe	Mn	Al	Ni	P	Pb	Sb	Si	Sn	Zn
Copper Cu-ETP	C11000	99.9	0.0	0.001	0.001	0.002	0.001	0.0	0.0	0.030	0.002	0.000	0.008	0.0	0.0
Red Brass CuZn15	C23000	85.7	0.001	0.001	0.001	0.001	0.001	0.0	0.001	0.001	0.001	0.001	0.010	0.001	14.3.
Yellow Brass CuZn37	C27400	63.2	0.001	0.001	0.001	0.001	0.001	0.001	0.06	0.001	0.004	0.001	0.008	0.0	36.7
Phosphor Bronze CuSn6	C51900	94.1	0.006	0.002	0.0	0.001	0.001	0.016	0.01	0.222	0.038	0.001	0.002	5.5.	0.1
Nickel-Aluminium Bronze CuAl10Ni5Fe4	C63000	82.2	0.03	0.001	0.002	3.5.	0.6	8.9.	4.6.	0.003	0.004	0.001	0.009	0.03	0.1
Cupronickel CuNi10Fe1Mn	C70600	87.8	0.001	0.001	0.001	1.5.	0.6	0.001	10.0	0.004	0.002	0.001	0.005	0.01	0.1
Nickel silver CuNi18Zn20	C75200	63.1	0.001	0.001	0.001	0.027	0.12	0.001	17.9.	0.001	0.001	0.008	0.001	0.001	18.9.
Nickel silver CuNi12Zn20	C75700	64.7	0.001	0.001	0.001	0.009	0.25	0.001	12.0	0.002	0.001	0.001	0.001	0.002	23.4.
Stainless Steel	S30400	Fe 68.8, C 0.07, Cr 19, Mn 2, N 0.1, Ni 10, P 0.045, S 0.015, Si 1													

* UNS (Unified Numbering System).

**Table 2:** Formulas of fitted functions.

Suspension Version	TSB		NaCl	
Bacteria, metallic material	R^2^	fitted function (x = time in minutes)	R^2^	fitted function (x = time in minutes)
EC, Cu	0.997	y = 7,539,362.8 x^−0.007x^	1	y = 2,000,000 e^−2.660x^
SA, Cu	0.999	y = 7,480,509.3 e^−0.038x^	0.857	y = 12,717,251 x^−0.014x^
EC, CuZn15	0.873	y = 9,839,313.2 x^−0.006x^	1	y = 8,500,000 e^−2.726x^
SA, CuZn15	0.862	y = 13,774,193 x^−0.006x^	0.998	y = 13,037,934 x^−0.030x^
EC, CuZn37	0.996	y = (4,509,969 − 14,996.8x)/(1 − 0.042x + 0.001x^2^)	1	y = 7,500,000 e^−0.414x^
SA, CuZn37	0.982	y = 10,395,963 x^−0.005x^	0.978	y = 11,082,599 x^−0.021x^
EC, CuSn6	1	y = 5,416,646.2 e^−0.097x^	1	y = 8,825,000 e^−2.726x^
SA, CuSn6	1	y = 11,333,353 e^−0.109x^	1	y = 21,999,999 e^−0.369x^
EC, CuNi10Fe1Mn	0.972	y = 5,559,082.2 x^−0.007x^	1	y = 7,100,000 e^−2.726x^
SA, CuNi10Fe1Mn	0.971	y = 7,562,494.3 e^−0.053x^	1	y = 7,999,117.9 e^−0.148x^
EC, CuNi12Zn24	0.965	y = 5,223,933.5 x^−0.002x^	1	y = 4,018,201.9 x^−0.029x^
SA, CuNi12Zn24	0.987	y = 9,523,622.2 x^−0.005x^	0.995	y = 9,585,646.5 x^−0.023x^
EC, CuNi18Zn20	0.951	y = 3,871,243.3 e^−0.013x^	1	y = 10,000,000 e^−0.504x^
SA, CuNi18Zn20	0.996	y = 4,816,607.3 e^−0.133x^	0.984	y = 1,246,559.1 x^−0.014x^
EC, CuAl10Ni5Fe4	0.817	y = 12,483,105 x^−0.002x^	1	y = 3,950,000 e^−2.660x^
SA, CuAl10Ni5Fe4	0.806	y = 4,625,374.2 x^−0.001x^	0.999	y = 19,999,988 e^−0,350x^
EC, Stainless steel	0.995	y = 12,787,135 x^−0.0002x^	0.908	y = 9,518,771.8 e^−0.005x^
SA, Stainless steel	0.924	y = 4,156,727.5 x^−0.001x^	0.886	y = (2,472,541.6 + 178,879.83x)/(1 − 0.014x + 0.0003x^2^)
